# Assessment and management of secondary bacterial infections complicating Mpox (Monkeypox) using a telemedicine service. A prospective cohort study

**DOI:** 10.1177/09564624231162760

**Published:** 2023-03-15

**Authors:** Samuel Moody, Thomas Lamb, Eleri Jackson, Alison Beech, Nabihah Malik, Leann Johnson, Nathan Jacobs

**Affiliations:** 1 5293 Manchester University NHS Foundation Trust, North Manchester General Hospital, Manchester, UK; 2 Centre of Tropical Medicine and Global Health, Nuffield Department of Clinical Medicine, 6396 University of Oxford Oxford, UK; 3 Lao-Oxford-Mahosot Hospital-Wellcome Trust Research Unit, Vientiane, Lao People’s Democratic Republic

**Keywords:** Monkeypox, mpox, cellulitis, telemedicine, secondary bacterial infection

## Abstract

**Introduction:**

During spring 2022, an outbreak of Monkeypox (mpox) emerged as an infection of concern in Europe. Due to the overlapping clinical features of mpox and bacterial infections, diagnosis of concomitant bacterial infection is challenging. In this prospective cohort study, we report the incidence, severity, and progression of patients with secondary bacterial infection complicating mpox infection.

**Method:**

Data were collected via a bespoke mpox telemedicine service provided by Infection services at North Manchester General Hospital, UK. A diagnosis of secondary bacterial infection was based on the history (balanitis, surrounding erythema, purulent discharge and nasal ulceration) and review of patient-collected medical photography. Patient were reviewed face-to-face where necessary.

**Results:**

Secondary bacterial infection was diagnosed in 15 of 129 (11.6%) patients with mpox. Three patients with secondary bacterial infection (3/129, 2.3%) required admission to hospital and one patient underwent surgical debridement. Median healing (thus, isolation) times were longer in those with bacterial infection.

**Discussion:**

In this prospective cohort study of patients with mpox, secondary bacterial infection was infrequent and predominantly mild. The virtual ward and telemedicine follow up allowed for the prompt recognition of secondary bacterial infections and timely antibiotic administration. Due to concerns regarding nosocomial transmission, mild clinical course and limited inpatient bed capacity, we believe this model of outpatient management for mpox (Clade II B.1 lineage) could be replicated in other low risk populations where suitable home isolation facilities exist.

## Introduction

In May 2022, the United Kingdom Health Security Agency (UKHSA) confirmed the first case of mpox (monkeypox) in the UK in a gentleman who had recently travelled to an endemic area. Following this case, the UK and other European countries saw a rapid rise in mpox cases without epidemiological link to endemic countries. As of 16 September 2022, there have been 3439 confirmed cases in the UK, with the origin of the current outbreak under investigation.^1^ Whole genome sequencing of the current circulating mpox virus has identified the current outbreak to be phylogenetically distinct and has been labelled as Clade IIb, with the majority of sequenced cases lineage B.1 or descendants.^1^

In the context of the current outbreak, men who have sex with men (MSM) have been the greatest affected sub-population.^1^ Emerging evidence details a plausible sexual route of transmission following the identification of the mpox virus in semen.^2^ Infection is characterised by a viral prodrome, pyrexia, lymphadenopathy, and mucocutaneous lesions (frequently affecting the genitals and perianal region); common complications include pharyngitis, penile oedema and proctitis^3–5^; Rarer sequelae include encephalitis, pneumonitis, keratitis and secondary bacterial infection.^4,6^ In the current outbreak of mpox, low case fatality has been seen globally and at the time of publishing, there have been no deaths reported in the UK.^1,6^

Due to the overlap of signs and symptoms of mpox infection mimicking that of bacterial infection, it is possible that this results in delays to diagnosis of bacterial infection- or conversely, that some patients are unnecessarily treated with antibiotic therapy. In this study, we aim to describe the frequency, severity, and evolution of mpox complicated by bacterial infections.

## Methods

The study was conducted by the Infectious Diseases (ID) department at Manchester University NHS Foundation Trust, UK. Patients that presented with a clinical syndrome consistent with mpox and met the UKHSA guidelines for possible/probable infection, were tested at the presenting organisation (most typically the local sexual health service). Patients were screened to identify whether they were clinically suitable for outpatient management and had an appropriate location to isolate. All patients were referred to the ID virtual ward and patients that tested negative were de-isolated and discharged from mpox follow up. Antibiotics were not prescribed routinely.

The virtual ward had a dedicated 24-h telephone line and secure messaging service to exchange photographs. Clinical staff initiated communication with patients on a daily basis; As mpox lesions improved contact was reduced to every other day. A diagnosis of secondary infection was based on the history (balanitis, surrounding erythema, purulent discharge and nasal ulceration) and review of patient-collected medical photography. If necessary, patients were assessed face-to-face using appropriate personal protective equipment to enable physical examination and the collection of sampling for microbiological culture. Data were collected prospectively and recorded into a live secure database.

## Results

As of 26 September 2022, 339 patients met the testing criteria stipulated by UKHSA and were tested for mpox using molecular polymerase chain reaction (PCR). Of these, 129 (38.1%) tested positive and were followed up on the virtual ward, the remaining 210 patients were deisolated and discharged back to the testing clinician. Two patients (2/129, 1.6%) were deemed to have inappropriate accommodation for home isolation were temporarily housed in a local hotel during their isolation period. Patients were accepting of the telemedicine consultation service and engaged well with text and telephone communication. Three patients (3/129, 2.3%) discontinued communication and were lost to follow up. Each of these three patients had recovered from their acute illness and were awaiting the complete healing of lesions before deisolation. No recognisable themes were identified with these patients to predict patients at risk of disengagement. These patients were notified to the local UKHSA health protection team.

**Figure 1.**
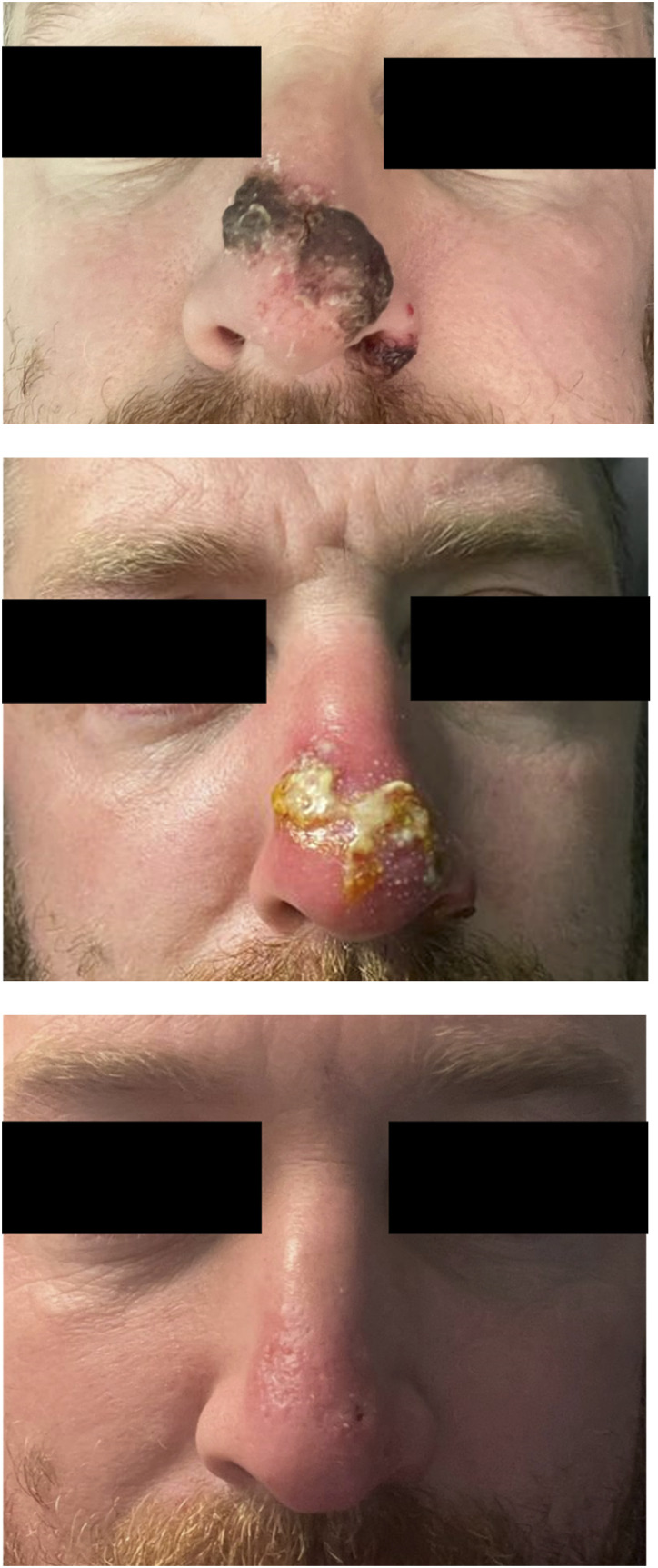
The evolution of bacterial infection to the nose of patient 11, with photos at day −1, day 5 and day 13 in relation to the date of his positive mpox swab of infection.

**Figure 2.**
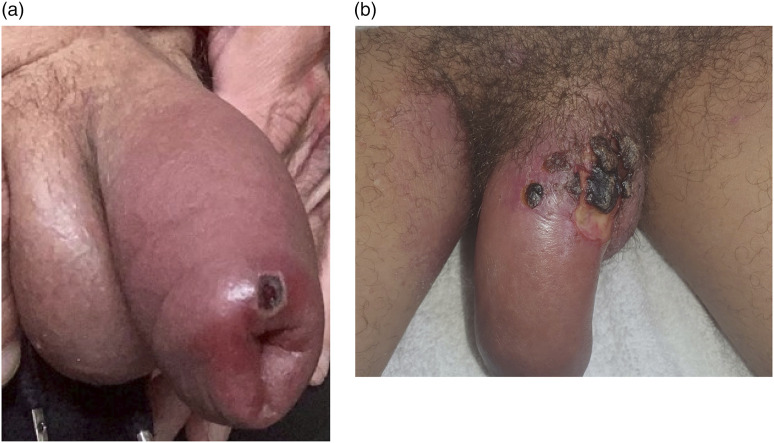
(a) Marked balanitis and erythema related to a mpox lesion on the penis in patients 6 and 12, respectively. The area of necrosis evident in (b) sloughed off without surgical intervention. The subsequent delayed healing resulted in a long period of isolation (38 days).

Based on clinical assessment, 15/129 (11.6%) of positive cases were treated for secondary skin and soft tissue bacterial infection. Eight patients were diagnosed with concomitant bacterial infection at presentation and a further seven were diagnosed during follow up. The demographic data, infection details and outcome for 12 patients that provided written informed consent are described in Table 1. All cases were male with a median age of 41 (IQR 34–50) years old. Three patients (20.0%) required admission to hospital for management of their bacterial infection for a duration of 2, 5 and 6 days respectively. One case required debridement under local anaesthesia and all cases made a full recovery. No patients were administered antiviral treatment. The median (Inter quartile range (IQR)) duration of isolation from date of positive test to de-isolation was 17.0 (14.4–22.0) days in cases diagnosed with bacterial infection and 13.0 (9.5–18.0) days for those without bacterial complication, *p* value .014. Although total numbers of secondary bacterial infection were low, the age, co-morbidities and mpox lesion site did not differ significantly to patients without secondary bacterial infection. Results of other sexually transmitted infections was not available as these investigations were processed under another, anonymised hospital record. Figure 1 provides photographic images of the extent, and resolution, of secondary bacterial infections affecting the nose. Figure 2 provides images of genital bacterial infections, with balanitis and cellulitis.

**Table 1.** A summary of patient demographics, infection site, bacteriology, treatment and outcome for patients treated for secondary bacterial infection complicating mpox infection.

Patient number	Age range (years)	Sex	Person living with HIV	Infection site	Microbiology	Antibiotic, route, dose, frequency, and duration	Duration of inpatient admission (days)	Duration of isolation (days)	Outcome
1	40–49	M	No	Penis	Group A haemolytic *Streptococcus*	Oral flucloxacillin 1000 mg QDS, 14 days	N/A	19	Full resolution
2	30–39	M	Yes	Upper leg, groin, and penis	Not sent	Oral flucloxacillin (dose and duration not recorded)	N/A	12	Full resolution
3	50–59	M	Yes	Scrotum	Not sent	Oral flucloxacillin 1000 mg QDS, 7 days	N/A	15	Full resolution
4	30–39	M	Yes	Groin	Not sent	Oral doxycycline 200 mg OD, 14 days	N/A	16	Full resolution
5	30–39	M	No	Penis	Not sent	Oral flucloxacillin, 1000 mg QDS, 7 days	N/A	14	Full resolution
6	50–59	M	No	Penis	Not sent	Oral co-amoxiclav, 625 mg TDS, 7 days	N/A	18	Full resolution
7	40–49	M	No	Scalp	Not sent	Oral flucloxacillin, 1000 mg QDS, 7 days	N/A	ND	Full resolution
8	30–39	M	No	Groin	Not sent	Oral Co-trimoxazole 960 mg BD, 7 days	NA	11	Full resolution
9	70–79	M	TBC	Penis	Not sent	Oral flucloxacillin, 1000 mg QDS (dose and duration not recorded)	N/A	20	Full resolution
10	50–59	M	No	Penis with balanitis	Group A haemolytic S*treptococcus*	Oral flucloxacillin 1000 mg QDS, 10 days	N/A	17	Full resolution
11	40–49	M	Yes	Nose	No significant bacterial growth	IV flucloxacillin 2000 mg QDS for 2 days, followed by Oral flucloxacillin for 8 days	2	15	Full resolution
12	20–29	M	No	Penis	Mixed anaerobes	IV flucloxacillin 2000 mg QDS for 4 days, followed by Oral flucloxacillin for 6 days Further course of co-amoxiclav 625 mg TDS for 7 days	5	38	Full resolution

M: Male; QDS: four times per day; TDS: three times per day; BD: twice daily; OD: once daily; mg: milligram; N/A: not applicable.

## Discussion

In this prospective cohort study of patients with mpox, secondary bacterial infection was infrequent and predominantly mild. Three of the 129 (2.3%) patients with confirmed mpox infection required admission to hospital for intravenous antibiotics. All bacterial infections had resolved on follow up and healed with minimal scarring and only one patient required surgical debridement. Patients engaged well with the virtual ward and telemedical service, and bacterial infections were identified and addressed promptly. The successful engagement of patients in the telemedicine service was likely influenced by the young age of patients, limited comorbidities, high rate of smart phone ownership and few patients (2/129, 1.6%) lacking suitable self-isolation facilities. The positive patient feedback and the favourable patient outcomes, support the role of home isolation and outpatient management during this current mpox outbreak of Clade II B.1 lineage.

The rapid evolution of this mpox outbreak necessitated a decision to manage patients at home with telemedicine support at short notice. The bespoke telemedicine service was delivered through a mobile phone and was supported by a confidential virtual ward using electronic records. Follow up care was delivered by the ID team with one doctor on rotation overseeing the management of 10–15 patients per day at any one time. Although the delivery of this service placed some additional resource strain upon the department, this was deemed significantly superior to admitting patients to hospital with the associated risks of nosocomial and healthcare worker transmission. Furthermore, patients preferred management at home where possible. Implementing this outpatient management protocol with telemedicine support can be adapted to a range of healthcare environments but should be tailored to the resources available. Consideration should be made as to whether patients can isolate alone within their normal place of residence.

Secondary bacterial infection was most frequently observed in the genital region (12/15 cases, 80.0%), reflecting the anatomical site with greatest number of mpox lesions. Due to the overlapping clinical features of fever, pain, and regional lymphadenopathy in patients with mpox, it is challenging to diagnose secondary bacterial infection. In addition, it is likely that attempts are made to minimise patient contact, resulting in an over treatment of secondary bacterial infection. In this study, additional clinical features that provoked suspicion and treatment of bacterial infection included the presence of surrounding erythema, marked balanitis/localized oedema, purulent exudate, and ulceration (Figures 1 and 2).

Bacterial infection was most marked in the two cases of secondary bacterial infection affecting the nose (Figure 1), as previously reported in the literature.^7^ In one patient, there were no other mpox lesions other than those affecting his nose. In this case, there was a delay of 2 days under the care of the ENT team before an epidemiological link was established with the current outbreak. This resulted in delays to testing for mpox and implementation of infection prevention and control precautions, and the delay in clinical suspicion resulted in the potential for nosocomial transmission. The coalescence of lesions affecting the nose with marked ulceration, are atypical for mpox lesions at other anatomical sites. Thus, mpox should be considered as a differential diagnosis of unexplained ulceration of the nose and prompt careful questioning to illicit an epidemiological exposure.

Mpox lesions secondarily infected with bacteria took longer to heal, crust over and for the scab to fall off, which was reflected by the longer median period of isolation for patients with secondary bacterial infection (17.0 days) compared to patients without secondary bacterial infection (13.0 days). In patient 12, the prolonged period of isolation (38 days) and associated stigma contributed to reported loneliness and poor mental well-being. This has been observed in other patients undergoing prolonged isolation^3^ and should be prospectively addressed during outpatient follow up to enable early recognition and referral to mental health support.

In this study no patients received antiviral therapy for mpox. There are case reports of good clinical outcomes following the use of antiviral treatment such as tecoviramat or cidofovir for mpox,^3,8^ and large scale randomised controlled trials are in progress.^9^

## Conclusion

The successful implementation of virtual ward and telemedicine follow up allowed for the prompt recognition of secondary bacterial infections and timely antibiotic administration. Due to concerns regarding nosocomial transmission and limited inpatient bed capacity, we believe this model of outpatient management for mpox (Clade II B.1 lineage) could be replicated in other low risk populations where suitable home isolation facilities exist. Particular attention should be made to patients with facial lesions and the impact of diagnostic stigma and isolation on mental well-being.

## References

[1] Investigation into monkeypox outbreak in England: technical briefing 8: UK Health Security Agency; 2022, https://www.gov.uk/government/publications/monkeypox-outbreak-technical-briefings/investigation-into-monkeypox-outbreak-in-england-technical-briefing-8

[2] Heskin J Belfield A Milne C , et al. Transmission of monkeypox virus through sexual contact - A novel route of infection. J Infect 2022; 85(3): 334–363. 10.1016/j.jinf.2022.05.028 PMC9534114 35659548

[3] Adler H Gould S Hine P , et al. Clinical features and management of human monkeypox: a retrospective observational study in the UK. Lancet Infect Dis 2022; 22(8): 1153–1162. 35623380 10.1016/S1473-3099(22)00228-6 PMC9300470

[4] Patel A Bilinska J Tam JCH , et al. Clinical features and novel presentations of human monkeypox in a central London centre during the 2022 outbreak: descriptive case series. Bmj 2022; 378: e072410. 35902115 10.1136/bmj-2022-072410 PMC9331915

[5] Girometti N Byrne R Bracchi M , et al. Demographic and clinical characteristics of confirmed human monkeypox virus cases in individuals attending a sexual health centre in London, UK: an observational analysis. Lancet Infect Dis 2022; 22(9): 1321–1328. 35785793 10.1016/S1473-3099(22)00411-X PMC9534773

[6] Thornhill JP Barkati S Walmsley S , et al. Monkeypox Virus infection in humans across 16 Countries - April-June 2022. N Engl J Med 2022; 387(8): 679–691. 35866746 10.1056/NEJMoa2207323

[7] Moschese D Giacomelli A Beltrami M , et al. Hospitalisation for monkeypox in Milan, Italy. Travel Med Infect Dis 2022; 49: 102417. 35934310 10.1016/j.tmaid.2022.102417 PMC9760088

[8] Labate L Brucci G Ciccarese G , et al. Nasal monkeypox virus infection successfully treated with cidofovir in a patient newly diagnosed with HIV. Int J Std AIDS 2022; 34(3): 208–210. 36520997 10.1177/09564624221141152

[9] Placebo-controlled randomised trial of tecoviramat in non-hospitalised monkeypox patients (PLATINUM). ISRCTN trial registry identifier:17461766. DOI: 10.1186/ISRCTN17461766 10.1186/ISRCTN17461766.

